# A Rare Cause of Urinary Retention Refractory to Conventional Measures: Bladder Fungoma

**DOI:** 10.7759/cureus.32024

**Published:** 2022-11-29

**Authors:** Emre B Sahinler, Orhun Sinanoglu, Erhan Erdogan, Yavuz Karaca

**Affiliations:** 1 Urology, Sancaktepe Sehit Prof. Dr. Ilhan Varank Research and Training Hospital, Istanbul, TUR

**Keywords:** lower urinary tract symptoms, urinary tract infection, candida infection, bladder, fungus ball

## Abstract

Funguria is a rare condition in healthy populations but in immunocompromised patients, it is occasionally seen and can cause opportunistic urinary tract infections. *Candida albicans* is the most commonly isolated pathogen in fungal urinary tract infections. The standard treatment for fungal urinary tract infection is anti-fungal therapy. Sometimes when severe funguria is present, a rare entity called a fungus ball can form in the urinary tract and surgical excision may be needed for eradication. In this report, we present a 76-year-old male patient who was admitted to our clinic for anuria for two days. The patient was catheterized transurethrally and saline irrigation was performed. *Candida albicans* was isolated from the urine. Ultrasound showed a 4 x 2cm fungus ball in the bladder. With the open surgical removal of the fungus ball and anti-fungal therapy with fluconazole, the patient was discharged without any complications. We emphasize that in patients with risk factors, abnormal imaging, and funguria resistant to anti-fungal therapy, fungus ball may be present and surgical removal is the standard approach.

## Introduction

Urinary tract infections (UTIs) are among the leading causes of medical care seeking. *Escherichia coli* is the most commonly seen pathogen in UTIs [1]. The prevalence of fungal UTIs has been rising in recent decades. Fungi are currently isolated in 7-8% of urine cultures [2]. Uncontrolled diabetes, immunosuppression, foreign bodies, long-term hospitalization, and use of broad-spectrum antibiotics are among the risk factors for fungal infection [3]. A large volume of residual urine, appropriate temperature, pH, and glucosuria favor the growth of *Candida albicans* in the urinary tract even in patients without risk factors [4]. A rare complication of candiduria is fungoma or fungus ball. The main location of fungoma is usually the kidney with the bladder being a very rare site [5]. In the bladder, fungoma presents a mobile, oval, and echogenic mass on ultrasound images resulting from the accumulation of long and wide pseudohyphae [6]. Fungomas are usually observed through ultrasound examination and seen as echogenic foci without demonstrable ultrasonic shadow [7]. There are only a few reports of primary fungal ball formation in the urinary bladder [5,8-10]. Fungus ball formation in the bladder can lead to obstruction and hydronephrosis and may require surgical intervention. In this case report, we present an elderly patient who had urinary retention due to a bladder fungus ball diagnosed by ultrasound examination and underwent open surgery for its removal.

## Case presentation

**Figure 1:**
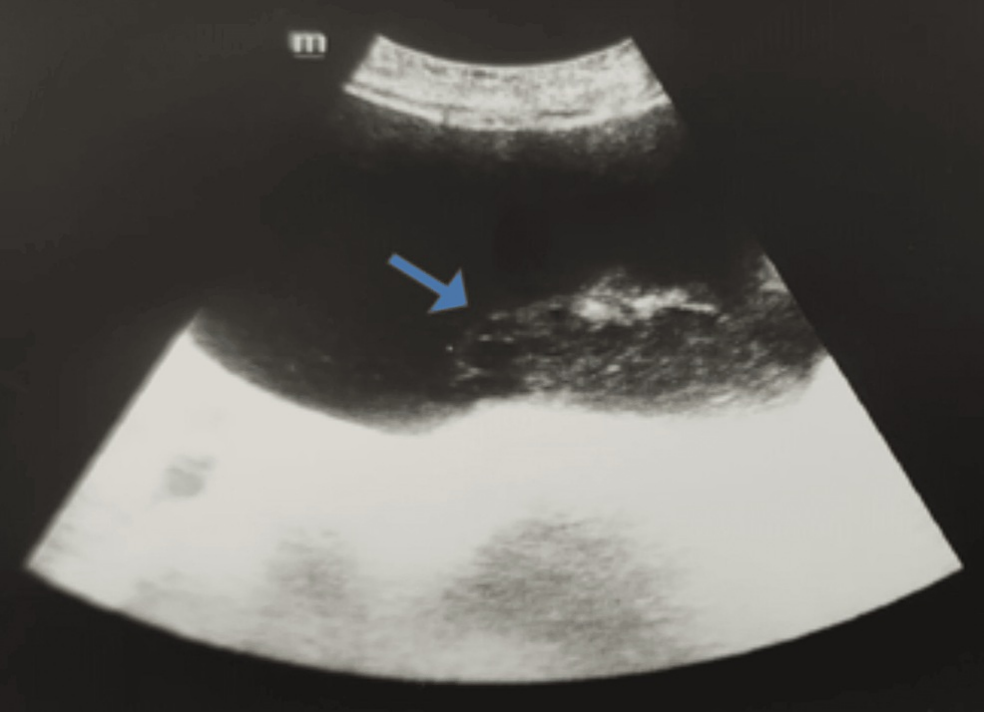
A fluctuating lesion with heterogenous echo 4 x 2 cm in size was detected on ultrasound

A 76-year-old male was admitted to our urology outpatient clinic due to anuria for two days. The patient had a history of controlled type 2 diabetes mellitus and coronary bypass surgery. His general condition was moderate with sub-febrile fever (37,6 °C). In physical examination, the bladder was palpable suprapubically due to acute urinary retention. In laboratory tests, his white blood cell (WBC) count was 12.4 10^3^/uL (high), C-reactive protein (CRP) level was 34 mg/L (high), fasting glucose level was 147 mg/dl (high), and creatinine level was 1.47 mg/dl (high). Other results were normal. The patient was catheterized by a three-way transurethral catheter. Bladder washouts via a transurethral catheter with saline were performed. The patient's transurethral catheter was changed several times, but the outflow got blocked in a short while. Cloudy whitish urine was present. *Candida albicans* was isolated from the urine. Systemic antifungal therapy (intravenous fluconazole 200mg/day) was initiated. Ultrasound (US) imaging showed high-grade hydroureteronephrosis and a few simple cysts in both kidneys. Prostate volume was 63cc with intravesical protrusion. The bladder wall was thickened to nearly 4mm when distended. There was mild trabeculation in the bladder mucosa and there was no sign of diverticula. A fluctuating lesion with heterogenous echo 4 x 2 cm in size was detected on the bladder base (Figure 1). 

As renal function tests started to deteriorate (creatinine level rose to 2.5 mg/dl), surgical intervention was decided. Cystoscopy was performed under general anesthesia. A white mobile mass measuring about 4 x 2 cm was encountered at the bladder base. The lesion could neither be dissolved despite vigorous washing maneuvers with an Ellik evacuator nor resected with a resectoscope. For that reason, the operation was converted to open surgery. Through a supra-pubic incision, we entered the bladder. During exploration, whitish heterogeneous fluid was aspirated and then the bladder lumen was irrigated with diluted povidone-iodine solution. The bladder wall was seen intact. The wound was closed and a drain was placed. The urinary sample showed necrotic material containing *Candida albicans* hyphae and spores. Intravenous fluconazole therapy (200mg/day) was continued for seven days postoperatively. The patient was discharged on the seventh postoperative day after urinary catheter removal and spontaneous voiding with a sterile urine culture.

## Discussion

Fungi are isolated in <1% of urine cultures in the normal population but rise up to 5-10% of urine specimens in hospitalized patients. *Candida albicans* is the most common organism in fungal UTI [11]. Secretions from the prostate gland in men and periurethral glands in women are protective against fungal UTIs in healthy adults [12]. Additionally, normal flora of the genitourinary mucous membrane prevents fungal colonization [5]. Diabetes mellitus, immunodeficiency, use of broad-spectrum antibiotics, and presence of foreign bodies are risk factors for fungal UTI [5,13]. Hospitalized patients with an indwelling bladder catheter have the highest risk of acquiring yeast in the urine [11].

Fungus ball is a rare complication of fungal UTI. Reports concerning fungus ball formation in the bladder are very few in the literature [5,8-14]. Two risk factors for *Candida* infection were present in our patient: bladder outlet obstruction due to prostatic hypertrophy and diabetes mellitus. Fluconazole is the drug of choice for *Candida* cystitis with the exception of drug-resistant *Candida glabrata*, *Candida krusei*, and other less common resistant yeasts. In patients in whom fluconazole is contraindicated or fails despite maximum doses, other agents such as oral flucytosine and parenteral amphotericin B (AmB) or local AmB bladder instillations may be necessary [15]. Since *Candida albicans* was reported in the first cytological examination, we administered intravenous (IV) fluconazole, but it was only partially effective. The Infectious Disease Society of America 2016 guidelines strongly recommend surgical intervention in *Candida* UTI associated with fungus ball [16]. So we decided on transurethral dissolution and evacuation of fungal mass based on previous reports suggesting that a transurethral resection should be the first surgical choice [5,17]. When the fungus ball is large and solid, open surgery might be the right choice. As the transurethral intervention was unsuccessful in our case, open surgery was performed to evacuate the infectious content. Furthermore, if an invasive manipulation such as transurethral or open surgery is performed to remove the resistant bladder fungus, systemic antifungal therapy or local irrigation with saline or antifungal drugs should be administered beforehand to prevent fungemia [16].

## Conclusions

Bladder fungus ball is a rare complication of fungal UTI. Anti-fungal therapy is the first treatment option. Since fungus balls can cause a mass effect and lead to lower and upper urinary tract obstruction, the disintegration of this entity is usually a must. Bladder irrigation through a transurethral catheter is the first option. When the fungus ball is resistant to irrigation, surgery may be necessary. In our case, we performed open surgery and managed to eradicate the infection. We believe this report can help guide the way for future studies. 
